# Subjective time expansion with increased stimulation of intrinsically photosensitive retinal ganglion cells

**DOI:** 10.1038/s41598-018-29613-1

**Published:** 2018-08-03

**Authors:** Pei-Ling Yang, Sei-ichi Tsujimura, Akiko Matsumoto, Wakayo Yamashita, Su-Ling Yeh

**Affiliations:** 1 Department of Psychology, National Taiwan University, Taipei, Taiwan; 2 Faculty of Science and Engineering, Kagoshima University, Kagoshima, Japan; 3 Graduate Institute of Brain and Mind Sciences, National Taiwan University, Taipei, Taiwan; 4 Neurobiology and Cognitive Neuroscience Center, National Taiwan University, Taipei, Taiwan; 5 Center for Artificial Intelligence and Advanced Robotics, National Taiwan University, Taipei, Taiwan

## Abstract

Intrinsically photosensitive retinal ganglion cells (ipRGCs) contain photoreceptors that are especially sensitive to blue light. Nevertheless, how blue light and ipRGCs affect time perception remains unsolved. We used the oddball paradigm and manipulated the background light to examine whether and how blue light and ipRGCs affect perceived duration. In the oddball paradigm, participants were asked to judge the duration of the target (oddball), compared to that of the standard, with a two alternative-forced-choice procedure. When the background light was controlled to be either blue or red in Experiment 1, results showed that blue light led to longer subjective duration compared to red light. Experiment 2 further clarified the contribution of the ipRGCs. A set of multi-primary projector system that could manipulate the ipRGC stimulation were used, while the color and luminance of the background lights were kept constant throughout. Results showed that increased stimulation of ipRGCs under metameric background expanded subjective time. These results suggest that ipRGC stimulation increases arousal/attention so as to expand subjective duration.

## Introduction

In this study, we examined the role of a population of intrinsically photosensitive retinal ganglion cells (ipRGCs) on observers’ duration judgment. The ipRGCs, a newly discovered third type of photoreceptors^1,2^, are easily stimulated by blue light emitted from various commonly used electrical devices (e.g., cellphone, pad, computer, room light, etc.) and affect circadian rhythm^3^, one kind of timing mechanisms in our body that governs metabolic function and sleep-awake cycle. The ipRGCs have been identified in rats^1,4^, monkeys, and human beings^5^. These cells are sensitive to light peaking at 481~493 nm (blue light) and are responsible for the effect of blue light on circadian rhythm. Especially, ipRGCs project to suprachiasmatic nuclei (SCN), which is the endogenous biological clock that allows external signal (i.e., ipRGC signal) to mediate the circadian rhythm^6,7^. Further, SCN is also associated with percived duration^8,9^, the kind of time perception we are interested in the current study.

However, little is known about the relationship between ipRGCs and perceived duration, since most previous studies focus on how blue light (compared to other color lights) affects perceived duration. Also, the studies that investigated time perception under blue light have rendered inconsistent results. These inconsistent results may result from different amount of ipRGC stimulation (i.e., in different color lights used), tasks (i.e., time production), or target time intervals (i.e., sub- or supra-second). For example, Caldwell and Jones^10^ used a production task for 30 and 40 seconds, and found no difference in time perception between red, white, and blue lights. Gorn^11^ used a supra-second estimation and found that time perception was shortened under blue light compared to red or yellow light. Katsuura, Yasuda, Shimomura, and Iwanaga^12^ used a production task to measure supra-second time interval, manipulating the background light as blue or red. Their results showed no effect between the two background conditions, except that time perception was lengthened at 180 s duration under blue light compared to that under red light. Shibasaki and Masataka^13^ used a time comparison task under blue and red light, with the sub-second to supra-second time intervals, and found shortened time perception under blue light, but only for men.

Table 1 summarizes the methods and results of these studies. This table shows that relationships between blue light and time perception have been examined by applying various tasks and with different time scales. Time production task asks participants to produce a specific time interval. Time estimation task demands participants to evaluate length of durations and response on scales (i.e., 1 = “slow” and 9 = “fast”). Time comparison task requires participants to categorize target durations into short or long. The various time perception paradigms have their own pros and cons. For example, time production task is more straightforward for evaluating participants’ subjective duration, but its variance is greater than the other paradigms. Time comparison task also requests participants to remember the standard time interval for categorization and thus also involves working memory. As to the different time scales used, they ranged from 0.4 s to 180 s across sub-second and supra-second scales, and these two scales have been shown to involve different mechanisms^8,9^. Specifically, the supra-second level is correlated with high cognitive functions, while the sub-second level is correlated with sensation or automatic processing^14–16^.

**Table 1:** Summaries of studies on blue light effect on time perception.

Study	Method			Results: time perception under blue light compared to other lights
	Task	Time interval	Peak wavelength	
Caldwell *et al*. (1985)	Time production	30 s, 40 s	*Not Available*	No effect
Gorn *et al*. (2004)	Time estimation	17.5 s	*Not Available*	Shorter than yellow/red
Katsuura *et al*. (2007)	Time production	90 s, 180 s	Blue: 436 nm Red: 612 nm	Longer than red (180 s)
Shibasaki *et al*. (2014)	Time comparison	0.4 s~1.6 s	*Not Available*	Shorter than red (male)

Most importantly, comparing time perception under different color lights necessarily involves different ipRGC stimulation and yet the role of ipRGCs on time perception has not been systematically examined in past studies. To examine the role of ipRGCs in the effects of blue light on perceived duration, especially bearing in mind that the sub-second level is more sensation involved, in this study we used the oddball paradigm to investigate an effect of attention/arousal process that is related to ipRGC stimulations^17,18^ on time perception. The oddball paradigm used in Tse, Intriligator, Rivest, and Cavanagh^19^ was adopted. In the oddball paradigm, participants are required to judge the duration of a low-probability but salient stimulus (oddball) and compare it to a string of high-probability standard stimuli with a fixed duration. The performance of the oddball paradigm could be influenced by attention level^19^ or arousal level^20^, which is suitable for examing the relationship between ipRGCs and time perception. The oddball paradigm has advantages over the production task, including that information of parameters in psychometric function could be collected and confounding factors from motor responses and participants’ memory ability can be eliminated^21^.

**Figure 1:**
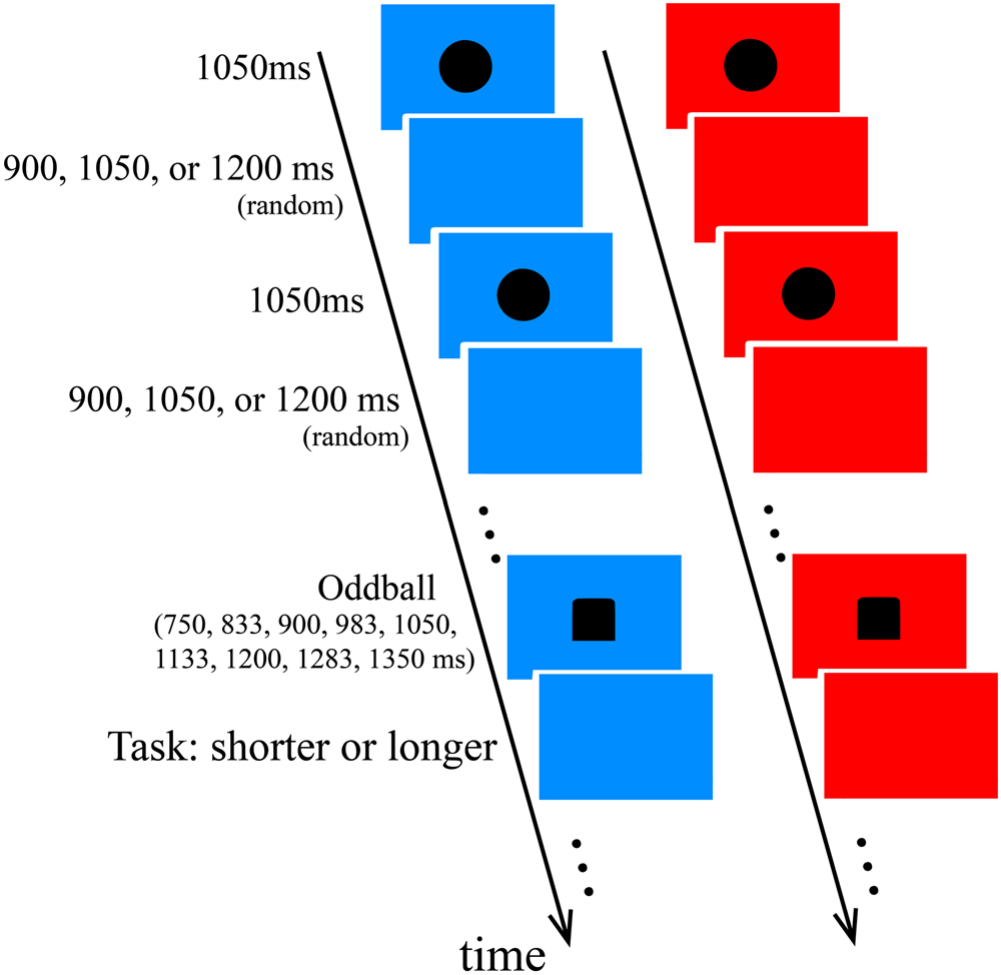
Procedure and stimuli used in this study. The duration of the standard stimuli (the circles) was fixed at 1050 ms, while that of the oddball (the square) varied and was chosen from one of the nine durations: 750, 833, 900, 983, 1050, 1133, 1200, 1283 and 1350 ms. Seven to 12 standards were presented randomly between two oddballs in the series. The inter-stimulus intervals were randomly chosen from one of the three durations: 900, 1050, and 1200 ms. The task was to judge whether the duration of the oddball was longer or shorter than that of the standards. In Experiment 1, the background could either be blue or red, while in Experiments 2 the background was gray. The displays are not plotted to scale.

By adopting the oddball paradigm, we were able to obtain the psychometric function and estimated the point of subjective equality (PSE) by comparing the judged duration of the oddball with that of the standards under different background conditions. In Experiment 1, the stimuli (standards and oddball) were presented in blue or red background (Fig. 1). As the explanations to the effect of blue light on perceived duration in past studies failed to consider the influence of ipRGCs, merely paying attention to the effect of background color, we examined the role of ipRGCs on perceived duration in Experiment 2. It is predicted that higher stimulation of ipRGCs would lead to greater stimulation of SCN and higher arousal level, then causing time perception to be lengthened.

## Experiment 1

In the oddball paradigm, we chose black circles as the standard stimuli and black square as the target, same as in Tsai and Yeh^21^.

### Methods

#### Participants

Eight healthy, male naïve volunteers (mean age = 23.25 years old) took part in Experiment 1. In this study, all participants had normal or corrected-to-normal vision, and if they wore glasses it was confirmed that their glasses did not contain blue light filters. Only male participants were recruited because time perception was only distorted with the male participants between blue and red^13^. They all gave informed consent before their participation. All experiments were approved by the Research Ethics Committee at National Taiwan University (NTU REC: 201505HS071) and conducted in accordance with applicable research subject guidelines.

#### Stimuli and Apparatus

The visual stimuli were controlled by E-Prime 1.0 and presented on a 19′′ CRT screen with 60 Hz refresh rate. The visual standards were black circles, and the oddball was a black square. The radius of the standards and the side length of the oddball were 1.7° visual angle, and they were presented on either blue light or red light background at the center of the screen. The duration of the standards was 1050 ms, and the duration of the oddball was randomly selected from one of the following nine durations with equal probability: 750, 833, 900, 983, 1050, 1133, 1200, 1283, and 1350 ms. The inter-stimulus intervals were randomly assigned as 900, 1050, or 1200 ms. Using a Spectroradiometer (PR650, Photo Research) to estimate the composition of blue light and red light background, the peak wavelength of the blue light was 452 nm (CIE xy color coordinate (0.14, 0.10)) and the peak of the red light was 628 nm (CIE coordinate (0.62, 0.34)). The luminance values in CIE 2006^22^ for blue light and red light was 9.51 cd/m^2^ and 5.99 cd/m^2^, respectively. We calculated the stimulation of ipRGCs based on the sensitivity curve of ipRGCs^23,24^ that has a peak wavelength of 493 nm, and stimulation of cones based on cone fundamentals at peripheral visual field in human^25,26^. The amount of ipRGC stimulation in the blue light condition was 42.16 times greater than in the red light condition. See Supplementary Materials 1 and 2 for cone and ipRGC stimulations and spectra for each condition.

#### Design

In Experiment 1, we adopted a 2 (background color: blue, red) × 9 (durations: 750, 833, 900, 983, 1050, 1133, 1200, 1283, and 1350 ms) within-subject factorial design. Background colors were counterbalanced between participants and each condition lasted for about one hour. In order to control the influence of circadian rhythm, participants performed the task at the same time of the day across the all the sessions. Each condition contained 378 oddballs in total, and each duration of oddball appeared 42 times in each condition.

#### Procedure

After dark adapted for five minutes, participants started the oddball task. Before the main task, participants needed to perform a practice session. The practice session had the same procedure with the main task. Seven oddball durations (750, 833, 900, 1050, 1200, 1283, and 1350 ms) randomly appeared once. In the main task, after 7~12 standard stimuli (randomly assigned between trials), an oddball would appear with one of the nine randomly assigned durations. In the task, participants were asked to judge whether the duration of the target stimulus (square) was longer or shorter than the standard stimuli (circles). They needed to respond immediately after the square disappeared, followed by a blank screen waiting for the response. No feedback was given as to the correctness of the response.

### Results

**Figure 2:**
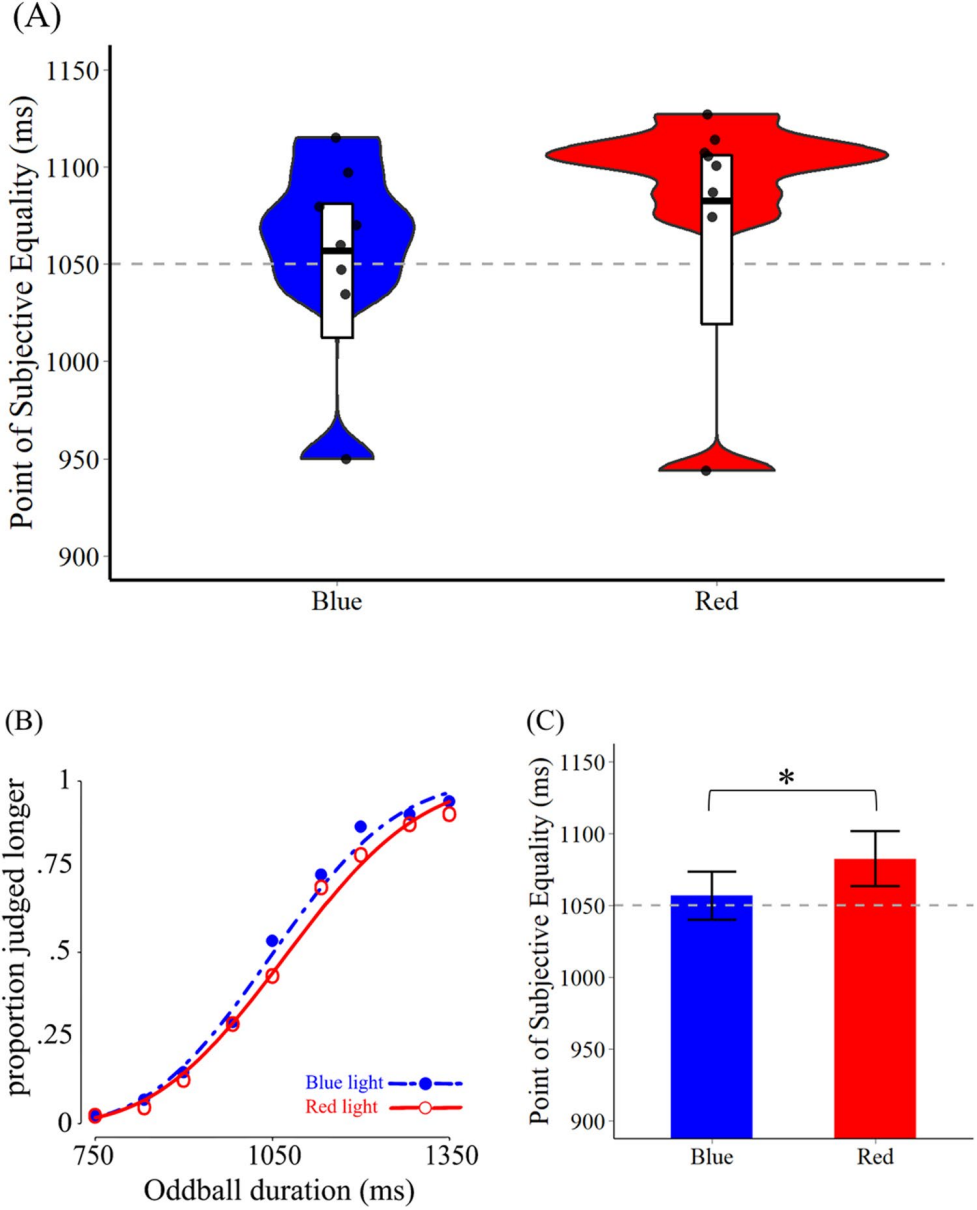
Results of Experiment 1. (**A**) The fitted PSE for blue and red light conditions in Experiment 2. The white bar in the middle indicates the bootstrapped 95% confidence intervals of each condition and the black horizontal line in the middle is the group mean. Each dot represents one participant’s fitted PSE value. The gray dashed line in the middle shows the duration of standard stimuli (1050 ms). (**B**) The group-averaged psychometric functions. (**C**) The PSE in the blue vs. red light conditions with bootstrapped 95% confidence intervals. The PSE under blue light was significantly smaller than that under red light, indicating a longer perceived duration under blue light than red light.

All analyses were executed using R^27^ and package ‟*modelfree*”^28^. We analyzed the three parameters: Point of Subjective Equality (PSE, the 50% chance that the duration was perceived as longer), Threshold, and Slope of psychometric functions for each of the participants and the group data (Table 2 and Fig. 2). All psychometric functions were fitted by the Weibull function. All the data can be found in the Data Availability Session below.

**Table 2:** Results of Experiment 1 and 2.

		Mean (SE)		
		PSE in ms	Threshold in ms	Slope
Exp1	Blue	1056.76 (16.65)	101.43 (11.33)	0.00244 (0.00035)
	Red	1082.51 (19.26)	109.76 (12.31)	0.00223 (0.00029)
Exp2	ipRGC High	1042.72 (15.89)	115.02 (9.78)	0.00196 (0.00015)
	Lightflux High	1063.66 (16.54)	102.85 (12.16)	0.00233 (0.00029)
	Control	1064.87 (18.91)	107.88 (11.57)	0.00216 (0.00021)

All parameters except PSE of the red light condition have passed the Shapiro-Wilk normality test. To deal with the non-normality issue of PSE data in the red light condition, a bootstrapping paired t-test was conducted for comparing the mean PSE from both conditions, using “*wBoot*” *R* package^29^ for 100000 iterations. The mean difference of PSE of both conditions was significant (PSE: bootstrapped mean = −25.79, 95% CI of the mean difference = [−51.88, −4.204], *p* = 0.0169). For comparing the threshold and slope of the two conditions, two paired t-test was conducted, respectively (Threshold: *t*(7) = −1.478, *p* = 0.183, 95% CI = [−21.663, 4.996]; Slope: *t*(7) = 1.284, *p* = 0.240, 95% CI = [−0.00017, 0.00058]). See Fig. 2C for the PSEs under the two (blue light vs. red light) conditions.

### Discussion

Our analyses in Experiment 1 yielded that perceived duration was lengthened under blue light compared to that under red light. Difference in color, cone, luminance, and ipRGC stimulation were possible factors contributing to this result. Thus, to clarify the role of ipRGCs in affecting perceived duration, in the next experiment, we created a pair of lights with the same color and luminance (i.e., the metameric pair) and manipulated different ipRGC stimulations to tease apart the effect of color and luminance^30,31^.

## Experiment 2

**Figure 3:**
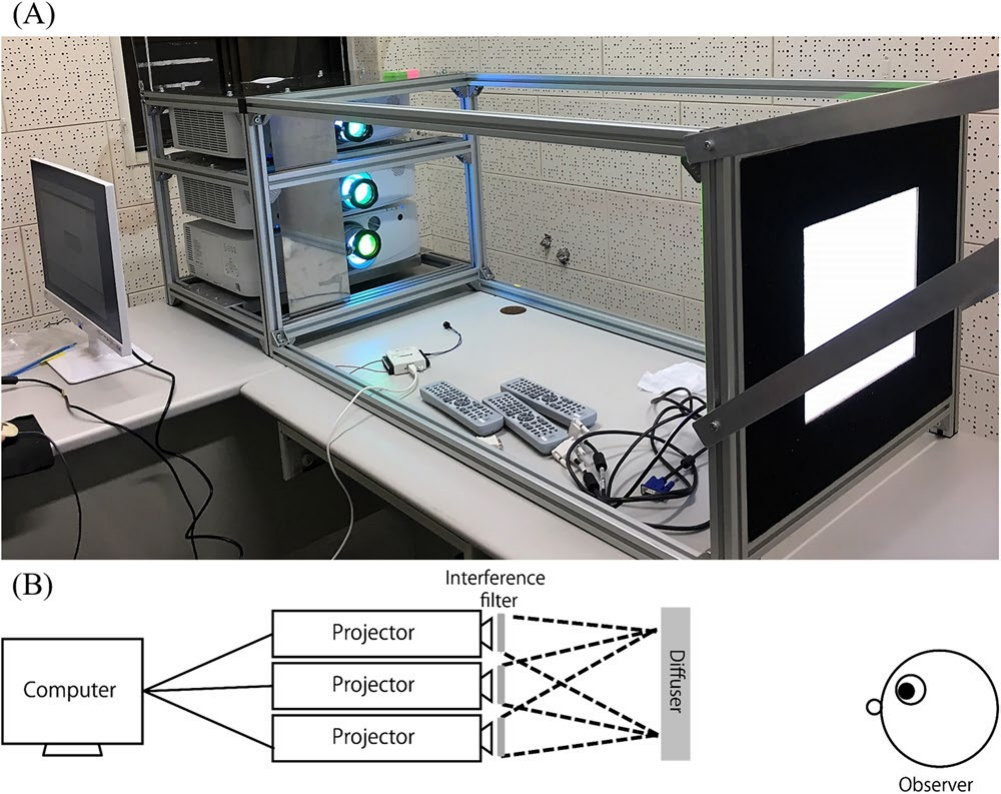
A set of multi-primary stimulation used in Experiment 2. (**A**) A multi-primary stimulator is a customized illumination system consisting of three projectors and interference filters which exploits a four-primary illumination system that enables independent stimulation of ipRGCs and the three types of cones. Three projectors on the left created a metameric background with different light components and projected to the screen (the light square on the right) in front of the participant, who sat at the right side that is out of the range of this photo. Black curtain covering the whole system was used in the experiment and it was removed for demonstration purpose here. (**B**) A schematic illustration of the experimental setting as described above.

In Experiment 2, we used a multi-primary stimulator (Fig. 3) that can independently manipulate stimulation of ipRGCs and the three types of cones^24,30,31^. This system allows us to further examine the influence of increased stimulation of ipRGCs and cones under the same color and luminance (i.e., metameric) backgrounds. By isolating each of these factors, it is expected to clarify the influences of each factors on time perception.

### Methods

#### Participants

Eight healthy, naïve volunteers (all males, mean age = 22.4 years old) took part in Experiment 2. Same participant inclusion criteria were used as in Experiment 1. They all gave informed consent before their participation. The experiment was approved by the Research Ethics Committee at Kagoshima University and all methods were performed in accordance with applicable research subject guidelines.

#### Stimuli and Apparatus

**Figure 4:**
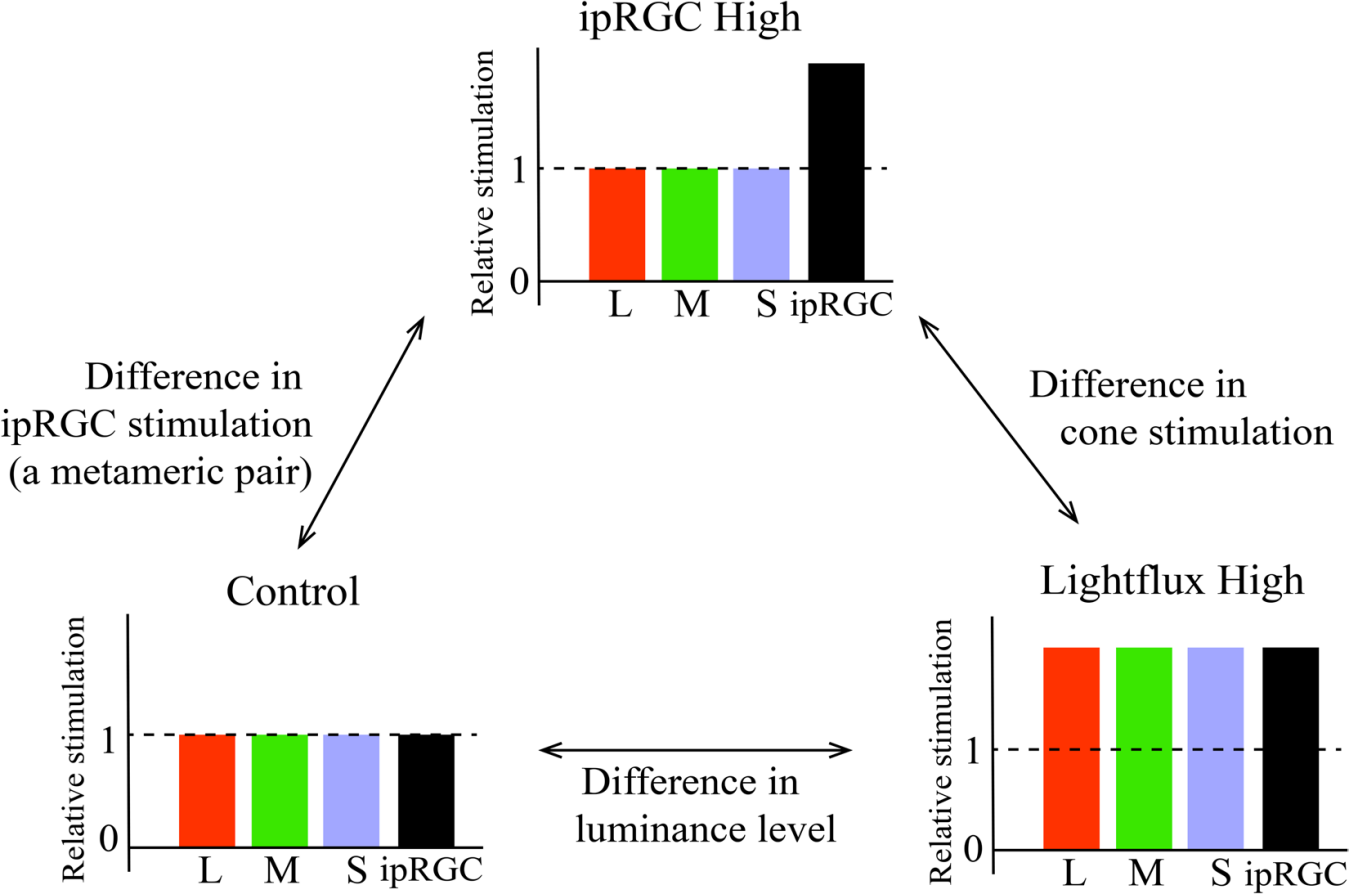
Three conditions in Experiment 2. Three conditions were manipulated, including ipRGC High condition, Control condition, and Lightflux High condition, and every two of the three conditions could be compared to examine the influence of ipRGC as well as cone stimulations (i.e., luminance). For the x-axis, “L”, “M”, and “S” refer to stimulations of the three kinds of cones: Long-wavelength, Middle-wavelength, and Short-wavelength cones.

Spatial arrangement and timing of the presentation of the test stimuli were the same as in Exp.1, except that the background color was always gray (CIE coordinate (0.40, 0. 38)) in all conditions. The observers were seated 81.0 cm in front of the display, which subtended 20.4° × 16.8° in visual angle. A multi-primary stimulator consists of three projectors and interference filters that enables independent stimulation of each photoreceptor class. The peak wavelengths of the four primaries were 455 nm, 530 nm, 580 nm, and 595 nm. We used this system to create three conditions, ipRGC High, Lightflux High and Control condition (Fig. 4). The luminance in the Control condition and the ipRGC High condition was 110 cd/m^2^ while the luminance in the Lightflux High condition was 228 cd/m^2^. The same calculation of cone stimulations was used as in Experiment 1. The display in the ipRGC High condition and that in the Control condition had the same luminance and color, indicating a metameric pair. The stimulation of ipRGCs in the ipRGC High condition was 2.1 times higher than that in the Control condition. By comparing the three conditions, we were able to tease apart the contribution of luminance, cone stimulation, and ipRGC stimulation to the duration judgment.

#### Design and Procedure

In Experiment 2, we adopted a 3 (condition: ipRGC High, Lightflux High, and Control) × 9 (duration: 750, 833, 900, 983, 1050, 1133, 1200, 1283, and 1350 ms) within-subject design. Each condition repeated three times. The other details were the same as in Experiment 1, except that the seven oddball durations in the practice session were randomly chosen from the nine durations (durations: 750, 833, 900, 983, 1050, 1133, 1200, 1283, and 1350 ms). All participants had been through five minutes light adaptation to a background in each condition.

### Results

**Figure 5:**
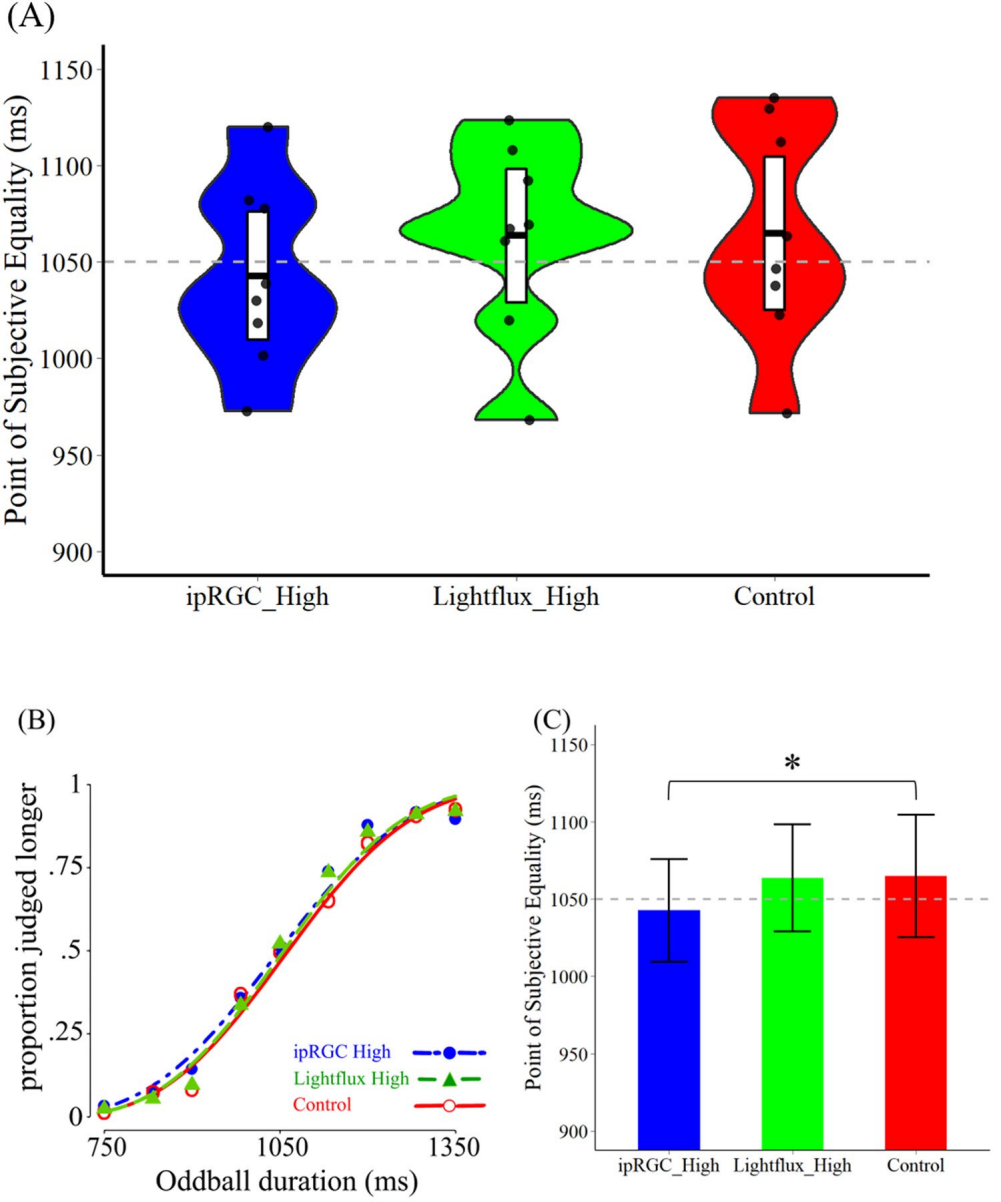
The results of Experiment 2. (**A**) The fitted PSE for all three conditions in Experiment 2. The white bar in the middle indicates the 95% confidence intervals of each condition and the black line is the group mean. Each dot represents one participant’s fitted PSE value. The gray dashed line in the middle shows the duration of standard stimuli. (**B**) The group-averaged psychometric function in the three conditions. (**C**) The PSE for each condition. The PSE in the ipRGC High condition was significantly lower than that in the Control condition, while no other differences were found between other pairs of conditions.

We analyzed the psychometric functions of eight participants (Fig. 5A), and the corresponding parameters. The data were fitted by Weibull model. The group-averaged data (Fig. 5B) of three conditions in Experiment 2 was summarized in Table 2. First, all the data for three parameters have passed the Shapiro-Wilk test for normality. In addition, we could do the further inference analysis with this data. We conducted the one-way repeated measure ANOVA for the three parameters, including PSE, threshold, and slope. Except for the significant main effect of PSE (PSE: *F*(2,14) = 4.929, *p* = 0.024, η_p_^2^ = 0.413) (Fig. 5C), both main effects of threshold and slope were not significant (Threshold: *F*(2,14) = 1.416, *p* = 0.275, η_p_^2^ = 0.168; Slope: *F*(2,14) = 1.640, *p* = 0.229, η_p_^2^ = 0.190). Then, the pairwise comparison showed that the significance of PSE came from the difference between ipRGC High condition and Control condition (Bonferroni corrected *p* = 0.021, 95% CI = [−40.515, −3.779]).

### Data Availability Statement

The R commands of psychometric function fitting and the raw data in this study are available from the link: http://epa.psy.ntu.edu.tw/data_repository/Time_perception_Data.

### Discussion

We used a set of multi-primary stimulator to increase stimulation of ipRGCs while color and luminance were kept constant and showed that increasing the stimulation of ipRGCs would affect perceived duration: Increased stimulation of ipRGC led to lengthened perceived duration compared to the Control condition. No difference between the other paired comparison of conditions indicates that cone stimulation might have an opposite effect to that of the ipRGCs. In the Lightflux High condition, since the cone stimulation increased in comparison with that in the ipRGC High condition, cones may inhibit the effects of ipRGCs’ lengthening perceived duration, resulting in no difference between the ipRGC High and Lightflux High conditions. That is, higher ipRGC stimulation would lengthen the perceived duration, while higher cone stimulation (i.e., luminance) might have the opposite effect of the ipRGCs.

## General Discussion

To reveal the role of ipRGCs on perceived duration and to resolve the controversy about the effect of blue light on time perception, we used an oddball paradigm as the task for time perception and manipulated different background conditions when judging the duration of the oddball. In Experiment 1, longer time perception was found under blue light compared to red light. In Experiment 2, we attempted to solely control the stimulation of ipRGCs with no change in luminance and color, using a multi-primary stimulator to create metameric conditions to understand the role of increased stimulation of ipRGCs and cones. Results yielded that perceived duration was lengthened for higher stimulation of ipRGCs, while the stimulation of cone may have opposite effect on perceived duration than the ipRGCs. Together, this study provided evidence to dissociate the effect of cones and ipRGCs in contributing the influence of blue light on time perception.

In past researches, the explanations of blue light on time perception mainly focused on the contribution of color properties, without clarifying the role of ipRGCs. Caldwell and Jones^10^ found no difference between blue light and other lights, using supra-second time production task, and indicated that their results were influenced by different characteristics of light, such as cold and warm. Gorn^11^ found time shrank under blue light, through conducting a supra-second time estimation task, and claimed that their results could be explained by different relaxing states induced by different colors. Katsuura *et al*.^12^ performed a supra-second time production task, and found time expanded under blue light only at 180 s duration. They listed various possible reasons, such as age, sex, arousal level, and ipRGC influences, but no further test was provided. Shibasaki and Masataka^13^ used a sub-second time comparison task, and found time shrank under blue light for men. They suggested that this was due to different implicit social meanings for blue than for red. Notably, previous researches did not clarify whether the changed time perception resulted from increased stimulation of ipRGC, or color perception, or both. In this study, we cannot distinguish the contribution of which components in Experiment 1 (cones, luminance, or ipRGCs) lengthened the perceived duration. However, the results from Experiment 2 strongly suggest that higher stimulation of ipRGC was responsible for lengthened perceived duration with the contribution from color excluded and luminance controlled. Although it seems that a larger PSE difference was found between the blue light condition and the red light condition (Experiment 1, mean = −25.75 ms, SE = 12.10 ms) than between the ipRGC High condition and the Control condition (Experiment 2, mean = −22.15 ms, SE = 5.49 ms), the difference between the two experiments was not statistically significant (*t*(9.7697) = −0.25, *p* = 0.805, 95% CI = [−35.35, 28.14]).

One might suspect that the subjective time expansion we found here might have been caused by rods rather than ipRGCs, since the peak wavelengths for spectral sensitivity curves of rods and ipRGCs are close to each other^23,31–33^. To minimize rod contribution in our setup, we used bright stimuli with luminance values between 110 cd/m^2^ and 228 cd/m^2^ in Experiment 2. The retinal illuminance was between 738 and 1533 scotopic trolands with a pupil size of 3.0 mm. According to Fuortes, Gunkel, and Rushton^34^, the incremental threshold for rod-based detection increased sharply at a light level above 100 scotopic trolands, suggesting that rods would saturate at the retinal illuminance we used in Experiment 2. Although we cannot completely rule out the possibility of rod intrusion, the involvement of rods in our Experiment 2 should be small and negligible^34–37^.

There are two possible explanations for time expansion with higher ipRGC stimulation: attention and arousal. First, duration judgment of the oddball could be affected by attention^19^. Lockley *et al*.^38^ found that participants showed higher sustained attention under blue light compared to green light. Also, the ipRGCs project to SCN, which controls circadian rhythm^6^ and is associated with attention^39^. In addition, blue light might increase the ipRGC stimulation and affect sustained attention, causing lengthened perceived duration. Second, it might be affected by arousal. We adopted the Scalar Timing Theory (STT) of time perception^40,41^ to explain the results we obtained. The variation of the PSEs obtained from the oddball paradigm has been shown to be contributed by the pacemaker component in the STT model^20^, and pacemaker can be accelerated by higher arousal state^42^. Moreover, higher ipRGC stimulation was correlated with greater arousal state, because the ipRGC neurons would transmit positive signals to SCN^38^. In addition, ipRGC neurons could modulate the SCN stimulation level^43^, further lengthening the perceived duration. Some behavioral experiments also showed the connection between circadian rhythm (controlled by SCN) and duration judgment; participants responded longer at night and morning than in the middle of the day in reproduction task^44^, a common time perception task asking participants to reproduce durations. Regardless of the exact underlying mechanism, both explanations (attention and arousal) need to base on the role of SCN and circadian rhythm. Through influencing the SCN, higher ipRGC stimulation would lengthen the duration judgment.

Notably, the perceived duration did not expand when the cones and ipRGC stimulation increased simultaneously, compared to the Control condition in Experiment 2. One possible explanation is that the influence on SCN between cones and ipRGCs could be opposite. According to Pilorz *et al*.^45^, the effect of delayed sleep onset, controlled by SCN, induced by blue light (470 nm) was established in melanopsin-normal mice but not in melanopsin-deficient mice. Moreover, Allen *et al*.^46^ showed that light adaptation ability was different between melanopsin-normal and -deficient mice, using receptor silent substitution. In ipRGCs, melanopsin serves a role as transducing light information to brain regions^1,4^. Hence, both studies support that some opposite interaction exists between cones and ipRGCs. In addition, here, higher ipRGC stimulation might lengthen perceived duration through increased SCN activation, while higher cone stimulation might shorten perceived duration and cancel out the lengthening effect caused by ipRGCs.

This is the first study using the oddball paradigm and manipulating the background light components to clarify the contributions of ipRGCs and color on time perception. This finding reveals how blue light and ipRGCs affect sub-second interval judgment. Future studies may further investigate the contribution of cones and ipRGCs to the attention and arousal process and conduct experiments on ipRGC-gene knockout mice to confirm the neural mechanism. The multi-primary stimulation used in Experiment 2, on the other hand, can easily manipulate the stimulation of ipRGCs and luminance without changes in color perception, and thus is suitable for conducting experiments in lab settings.

## Electronic supplementary material

supplementary material
