# Supplementary material for: Subjective time expansion with increased stimulation of intrinsically photosensitive retinal ganglion cells
Source: Sci Rep. 2018 Aug 3;8:11693. doi: 10.1038/s41598-018-29613-1 (PMC6076248; doi:10.1038/s41598-018-29613-1)

**Subjective time expansion with increased stimulation of intrinsically photosensitive retinal ganglion cells**

Pei-Ling Yang^1^, Sei-ichi Tsujimura^2^, Akiko Matsumoto^2^, Wakayo Yamashita^2^, and Su-Ling Yeh^1,3,4,5*^

**^1^**Department of Psychology, National Taiwan University, Taiwan

**^2^** Faculty of Science and Engineering, Kagoshima University, Japan

**^3^** Graduate Institute of Brain and Mind Sciences, National Taiwan University, Taiwan

**^4^** Neurobiology and Cognitive Neuroscience Center, National Taiwan University, Taiwan

**^5^** Center for Artificial Intelligence and Advanced Robotics, National Taiwan University, Taiwan

*Corresponding author

Su-Ling Yeh

Department of Psychology, National Taiwan University

No. 1, Sec. 4, Roosevelt Rd., Taipei 10617, Taiwan.

Tel：+886-2-3366-3955

E-Mail: suling@ntu.edu.tw

**Supplementary Material 1. Stimulation of cones and ipRGCs based on10-deg fundamentals (see main text for further details)**

| Experiment | Condition | L (cd/m^2^) | M (cd/m^2^) | S^#^ (cd/m^2^) | ipRGC^#^ (cd/m^2^) | Luminance (cd/m^2^) |
| --- | --- | --- | --- | --- | --- | --- |
| Experiment 1 | Blue | 5.35 | 4.16 | 70.72 | 40.97 | 9.51 |
|  | Red | 5.12 | 0.87 | 0.58 | 0.97 | 5.99 |
|  | Ratio of blue/red | 1.05 | 4.80 | 121.46 | 42.16 | 1.59 |
| Experiment 2 | ipRGC High | 79.12 | 30.70 | 65.01 | 83.95 | 109.82 |
|  | Lightflux High | 164.32 | 63.76 | 135.02 | 83.95 | 228.08 |
|  | Control | 79.12 | 30.70 | 65.01 | 40.42 | 109.82 |
|  | Ratio of ipRGC High/Control | 1.00 | 1.00 | 1.00 | 2.08 | 1.00 |
|  | Ratio of Lightflux High/Control | 2.08 | 2.08 | 2.08 | 2.08 | 2.08 |
|  | Ratio of ipRGC High/Lightflux High | 0.48 | 0.48 | 0.48 | 1.00 | 0.48 |
| Note: # We assumed that neither the S cones nor the ipRGCs affect the photopic luminance efficiency function (i.e., luminance), despite using photopic luminance units (cd/m^2^). Similar to S-cone stimulation (Boynton & Kambe, 1980), one ipRGC stimulation was defined as the level of ipRGC stimulation produced by an equal energy spectrum of luminance of 1 cd/m^2^. | | | | | | |

Reference: Boynton, R. M., & Kambe, N. (1980). Chromatic difference steps of moderate size measured along theoretically critical axes. *Color Research & Application*, *5*(1), 13-23.

**Supplementary Material 2. Spectra of stimuli used in this study**


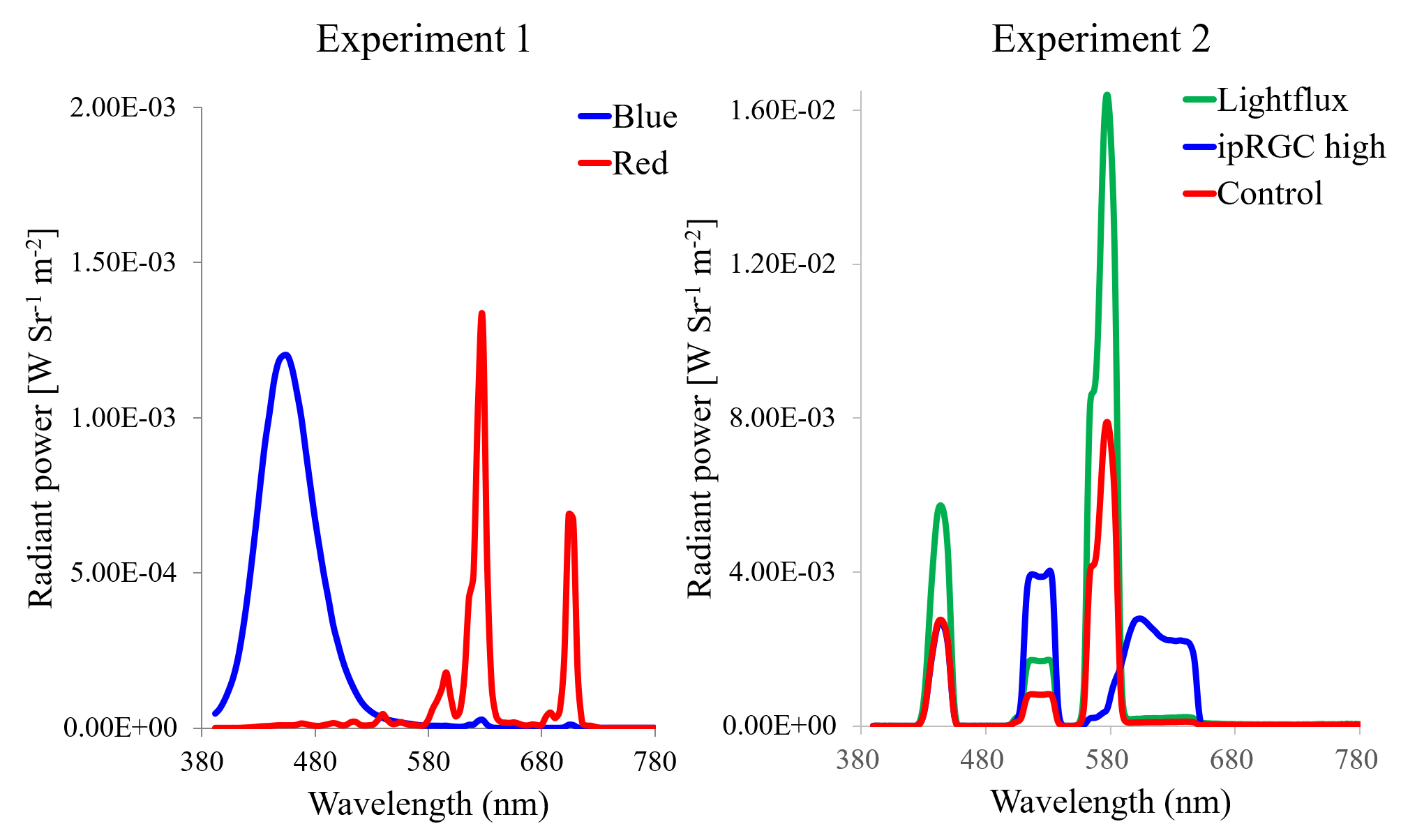

Supplement: Supplementary file 1 — supplementary material [file 41598_2018_29613_MOESM1_ESM.docx]
